# An Optimized Lock Solution Containing Micafungin, Ethanol and Doxycycline Inhibits *Candida albicans* and Mixed *C*. *albicans – Staphyloccoccus aureus* Biofilms

**DOI:** 10.1371/journal.pone.0159225

**Published:** 2016-07-18

**Authors:** Livia Lown, Brian M. Peters, Carla J. Walraven, Mairi C. Noverr, Samuel A. Lee

**Affiliations:** 1 Section of Infectious Diseases, New Mexico Veterans Healthcare System, Albuquerque, New Mexico, United States of America; 2 Division of Infectious Diseases, University of New Mexico Health Science Center, Albuquerque, New Mexico, United States of America; 3 Department of Prosthodontics, LSU Health Sciences Center, School of Dentistry, New Orleans, Louisiana, United States of America; 4 Department of Microbiology, Immunology and Parasitology, School of Medicine, LSU Health Science Center, New Orleans, Louisiana, United States of America; 5 Department of Clinical Pharmacy, University of Tennessee Health Sciences Center, Memphis, Tennessee, United States of America; 6 College of Pharmacy, University of New Mexico, Albuquerque, New Mexico, United States of America; Venenum Biodesign, UNITED STATES

## Abstract

*Candida albicans* is a major cause of catheter-related bloodstream infections and is associated with high morbidity and mortality. Due to the propensity of *C*. *albicans* to form drug-resistant biofilms, the current standard of care includes catheter removal; however, reinsertion may be technically challenging or risky. Prolonged exposure of an antifungal lock solution within the catheter in conjunction with systemic therapy has been experimentally attempted for catheter salvage. Previously, we demonstrated excellent *in vitro* activity of micafungin, ethanol, and high-dose doxycycline as single agents for prevention and treatment of *C*. *albicans* biofilms. Thus, we sought to investigate optimal combinations of micafungin, ethanol, and/or doxycycline as a lock solution. We performed two- and three-drug checkerboard assays to determine the *in vitro* activity of pairwise or three agents in combination for prevention or treatment of *C*. *albicans* biofilms. Optimal lock solutions were tested for activity against *C*. *albicans* clinical isolates, reference strains and polymicrobial *C*. *albicans-S*. *aureus* biofilms. A solution containing 20% (v/v) ethanol, 0.01565 μg/mL micafungin, and 800 μg/mL doxycycline demonstrated a reduction of 98% metabolic activity and no fungal regrowth when used to prevent fungal biofilm formation; however there was no advantage over 20% ethanol alone. This solution was also successful in inhibiting the regrowth of *C*. *albicans* from mature polymicrobial biofilms, although it was not fully bactericidal. Solutions containing 5% ethanol with low concentrations of micafungin and doxycycline demonstrated synergistic activity when used to prevent monomicrobial *C*. *albicans* biofilm formation. A combined solution of micafungin, ethanol and doxycycline is highly effective for the prevention of *C*. *albicans* biofilm formation but did not demonstrate an advantage over 20% ethanol alone in these studies.

## Introduction

*Candida albicans* is a major source of nosocomial bloodstream infections in the United States [1] and treatment can be complicated by the presence of indwelling medical devices, particularly central venous catheters [1–4]. Persistent infection of medical devices with *Candida spp*. can occur despite treatment, leading to recurrent infections [2]. Treatment of catheter-related infection may be challenging due to the formation of biofilms, which are dense communities of hyphal and yeast cells characterized by increased resistance to host immune responses and antifungal therapy [5–7]. *Candida* biofilms are up to 1000-fold more resistant to standard azole treatment [8] and up to 20 times more resistant to echinocandin treatment compared to susceptibilities when in planktonic form [9–11]. Increased resistance within biofilms is a multifactorial process; in early stages of biofilm formation, the expression of drug efflux pumps encoded by *CDR1*, *CDR2*, and *MDR1* is increased [12] and during later stages, the extracellular matrix may prevent antifungal drugs from reaching underlying cells [13]. The extracellular matrix has three essential polysaccharide components including α-mannan, β-1,3 glucan and β-1,6 glucan [14]. Resistance in *C*. *albicans* may also be due to mutations in drug targets or alterations in membrane sterol composition, metabolic salvage pathways [15–16] and a reduction in metabolic activity inherent to the inner biofilm cells [17]. Due to the limited presence of nutrients and oxygen in this environment, antimicrobials that depend on cell viability are rendered ineffective. Thus, biofilm-related infections in medical devices, including those caused by *C*. *albicans* are often difficult to treat.

In the event of a suspected catheter-related bloodstream infection due to *Candida* species, current Infectious Diseases Society of America guidelines recommend catheter removal in most cases [18]. However, central venous catheter removal and re-insertion can be associated with a number of serious and not infrequent complications, such as bleeding, arterial puncture, hematoma, and pneumothorax [19]. An investigational alternative to catheter removal is antifungal lock therapy (AfLT). AfLT, like antibacterial lock therapy, attempts to sterilize an infected catheter or medical device through prolonged exposure to a solution containing high concentrations of antifungals in conjunction with systemic antifungal therapy. Locally instilled lock solutions may contain up to 1000 times the concentration of an antimicrobial drug than that given systemically [4], thereby increasing the potential for microbial eradication.

A number of drug combinations have been tested *in vitro* as potential antimicrobial lock solutions, using the checkerboard technique. The checkerboard assay is designed to test the activity of multiple drugs in combination and has been a successful approach to determining *in vitro* combination efficacy, including evaluation of echinocandin-resistant *Candida* strains [20–21]. The checkerboard technique has uncovered several combinations that demonstrate synergistic activity, which is defined as an increase in the antimicrobial activity produced by a combination of drugs which is greater than the sum activity of each single drug alone [21]. Some drug combinations previously tested and which demonstrated synergistic activity include fluconazole combined with finasteride [22], doxycycline [23], terpenes [24], and others. However, a number of antifungal agents that may be used in a lock solution have shortcomings. For instance, resistance to fluconazole has been widely documented in biofilms and certain non-*albicans Candida* species [25–27] and higher concentrations of ethanol may compromise the integrity of polyurethane catheters (see discussion) [28].

Micafungin (MICA) is a potent fungicidal echinocandin with activity against *Candida spp*., *Aspergillus* and some other pathogenic molds [29]. Echinocandins are a class of antifungal drug that target cell wall synthesis by inhibiting 1,3-β glucan synthase [29]. Ethanol (EtOH) is an inexpensive sterilizing agent [30] with the advantage of reduced potential for resistance through its non-specific activity of protein denaturation [31]. Similarly, doxycycline (DOX) is a widely used broad-spectrum antibiotic [32] and has demonstrated *in vitro* antifungal activity in combination with fluconazole [23]. Tetracycline exerts antifungal activity though ribosomal inhibition in mitochondria, which are similar in structure to the bacterial 30S ribosomal subunit. Concentrations required for antifungal activity are much higher than used for gene induction [33]. As a component of an antifungal lock solution, DOX could potentially broaden the utility of the solution to include antibacterial activity, particularly against medically important bacteria such as *Staphylococcus aureus*. DOX has demonstrated both enhanced and antagonistic activity with the antifungal amphotericin B [34–35], suggesting that DOX may be useful in combination with other antifungals, although an antagonistic interaction is also theoretically possible.

*S*. *aureus* is a leading contributor to nosocomial bloodstream infections [1] with strong biofilm forming capabilities. One important review estimated that 27% of nosocomial *Candida* bloodstream infections were polymicrobial, and *S*. *aureus* was the third most common species co-isolated with *C*. *albicans* [36]. This review also reported a 36–57% incidence of candidemia-bacteremia co-infection, when the isolation of bacteria occurred 48 hours before or after *Candida spp*. isolation. *Staphylococcus* species represented 20% of these dual infections. An *in vivo* animal study demonstrated a synergistic increase in virulence when *S*. *aureus* and *C*. *albicans* were present together [37]. Specifically, a combination of 1/4 the lethal dose (50%) of *C*. *albicans* and 1/8 the lethal dose (50%) of *S*. *aureus* was enough to cause 100% mortality in a murine model. Additionally, *C*. *albicans* may confer vancomycin resistance to *S*. *aureus* via excretion of extracellular matrix [38]. The presence of a polymicrobial biofilm may complicate treatment by augmenting resistance to monomicrobial therapies; thus a lock solution with both antifungal and antibacterial activity for prevention and treatment may be advantageous.

In this study, we sought to determine the optimal combination of MICA, DOX and/or EtOH for use as an antifungal lock solution for the prevention and treatment of *C*. *albicans* biofilms *in vitro*. We further tested optimized solutions against *C*. *albicans*- *S*. *aureus* mixed biofilms. MICA, EtOH, and DOX were chosen because they have been shown to have strong antifungal activity against *C*. *albicans* and *S*. *aureus* [35, 39–42] and are clinically widely available. Our objective was to determine whether the potent antifungal activity of MICA combined with the activity of DOX would allow us to minimize the concentration of EtOH, thereby reducing the potential risk to the integrity of polyurethane catheters [30]. To establish an optimized antifungal lock solution, we performed two drug (2D) and three drug (3D) checkerboard assays as well as fungal colony regrowth assays. Optimized antifungal solutions were then tested for activity against additional *C*. *albicans* laboratory reference strains, two clinically-derived echinocandin resistant isolates as well as forming and mature *C*. *albicans*- *S*. *aureus* mixed biofilms. We demonstrate that the combination of 20% EtOH, 0.01565 μg/mL MICA and 800 μg/mL DOX does not improve the efficacy of the lock solution compared to ethanol alone at preventing the regrowth of forming and mature *C*. *albicans* monomicrobial biofilms in a silicone disk model at microbiologically relevant concentrations. Synergistic activity was only detected in combination with much lower concentrations of ethanol.

## Materials and Methods

### Microbial strains and growth conditions

*C*. *albicans* reference strains ATCC10231, ATCC14053 (fluconazole resistant) and ATCC24433 were purchased from ATCC (Manassas, VA). Clinically derived echinocandin resistant isolates 42379 and 53264 [43] were also studied in addition to the standard laboratory strain SC5314 [44]. All strains were grown and maintained at 30°C in yeast peptone dextrose (YPD, 1% yeast extract, 2% peptone, and 2% glucose, Becton, Dickinson and Company, Sparks, MD). Overnight cultures used in biofilm treatment, prevention and regrowth studies were re-suspended to a final density of 1.0×10^6^ cells/mL in RPMI-1640 media (Media Tech, Inc, Corning, NY). Concentrated (4X) RPMI media (20.8g RPMI-1640 with L-glutamine and 69g MOPS in 500mL H_2_O) was buffered to pH 7.0 with NaOH and stored at 4°C in a light-impermeable container. MICA was kindly provided by Astellas Pharma (Northbrook, IL); EtOH (100%) and DOX were purchased from Sigma-Aldrich (St. Louis, MO).

*S*. *aureus* strain M2 was used for all polymicrobial biofilm studies [45]. *S*. *aureus* was maintained as a glycerol stock at -80°C, streaked onto trypticase soy agar (TSA), and incubated at 37°C prior to use. A single colony was inoculated into 5 mL of trypticase soy broth (TSB) and incubated overnight at 37°C with shaking at 200 rpm. The following day, *S*. *aureus* was sub-cultured at a 1:100 dilution in fresh TSB and incubated at 37°C with shaking for three hours until the cells reached the early log phase of growth. Following growth, microbes were washed in phosphate buffered saline (PBS), counted on a hemocytometer, and adjusted to 1×10^7^ CFU/mL in 0.6X TSB containing 0.2% glucose (TSB-g). TSB-g was previously determined to be an optimal medium for supporting biofilm growth of both *S*. *aureus* and *C*. *albicans* [46–47].

### Antifungal activity assays

#### Preparation of drug solutions

All drug stock solutions were prepared in sterile ultra-pure water using 1:2 serial dilutions, in accordance with Clinical Laboratory and Standards Institute guidelines [48]. Each drug was prepared as a concentrated solution and then diluted in the wells of a 96-well plate by the addition of media, cells and other drug(s) to complete each antifungal lock solution. For use in 2D checkerboards, ten stock solutions (1:2 dilutions) of MICA were prepared at concentrations four times that of the final concentration. The highest concentration of MICA tested was 0.0313 μg/mL. DOX and EtOH stock solutions were prepared at twice the final concentration, which ranged from 1600 μg/mL to 3.125 μg/mL, and 40% to 0.625% (v/v), respectively. Each well was brought to a final volume of 200 μL. When DOX was used in combination with EtOH, working stocks of EtOH were prepared as described above, while DOX was prepared at four times its final concentration. Final DOX concentrations tested ranged from 800 μg/mL to 1.625 μg/mL.

All drug stocks used in 3D checkerboards were made at four times the final concentration tested and a single concentration of EtOH (5, 10, or 20%) was applied to every well in a single plate. The highest concentration of MICA tested was 0.0313 μg/mL with a total of twelve different concentrations utilized. DOX was used at eight different concentrations beginning with 800 μg/mL.

#### 2D checkerboard assays

An overnight culture of SC5314 was washed twice with PBS and re-suspended to a density of 4.0×10^6^ cells/mL in 4X RPMI. Media containing cells were added to a 96-well, flat bottomed, polystyrene micro dilution plate (Costar 3595, Corning Inc. Corning, NY) and diluted four-fold in the plate with single-drug stock solutions to create unique combination AfLT solutions. The plate was then incubated for 24 hours at 37°C. Each concentration of MICA was added to a column (1–10) while either DOX or EtOH were added to the plate in rows (1–7), producing a different combination of drug concentrations in each well. One column in the plate was reserved for positive (cells with media) and negative (media-only) control wells. Another column was reserved as a DOX or EtOH only control (single drug activity against *C*. *albicans* cells). One row was reserved as a MICA or DOX only control. Controls were diluted to the final concentration with water.

The antifungal activity of each solution against mature biofilms was then examined [49]. First, 4×10^6^ cells/mL in 4X RPMI were diluted to a final concentration of 1×10^6^ cells/mL in RPMI with sterile water, and allowed to incubate at 37°C for 24 hours. Mature biofilms were then washed three times with PBS before media and single-drug solutions were applied as described above. The plate was then incubated for an additional 24 hours before metabolic activity was assayed. Each 2D checkerboard (prevention and treatment) was performed independently three times.

#### 3D checkerboard assays

The arrangement of the 3D checkerboard assay was similar to that of the 2D. A separate control plate was necessary for calculating the activity of the three drugs in combination. The control plate included positive (cells with RPMI), negative (RPMI only), and single drug control wells. Every drug concentration tested in the checkerboard had three technical replicates performed to establish the activity of each drug concentration alone. Incubation conditions, times, and washes were consistent between 2D and 3D checkerboards. Each assay was repeated three times independently.

#### XTT reduction assay

The antifungal activity of MICA+DOX, MICA+EtOH and DOX+EtOH in the prevention and treatment of biofilm formation was assessed using the colorimetric XTT [2,3-bis-(2-methoxy-4-nitro-5-sulfophenyl)-2H-tetrazolium-5-carboxanilide] reduction assay as previously described [49]. Briefly, following incubation with an antifungal lock combination solution, each plate was washed three times with 200 μL PBS. Menadione was added to XTT solution and 100 μL of the resulting mixture was added to each well. The plate was then wrapped in aluminum foil and incubated for an hour and a half to two hours at 37°C. Absorbance was read in a plate reader at 490nm (BioTek model ELx808, Winooski, VT).

#### Antifungal activity of combination solutions against *C*. *albicans* isolates

Strains SC5314, ATCC10231, ATCC14053, ATCC24433, 42379, and 53264 [50–51] were resuspended to a concentration of 4×10^6^ cells/mL in 4X RPMI. Two optimized solutions were tested: (“OS 1”) 20% EtOH, 0.01565 μg/mL MICA, and 800 μg/mL DOX, and (“OS 2”) 10% EtOH, 0.0313 μg/mL MICA, and 800 μg/mL. Each strain was added to wells 1–12 of a designated row in two 96-well plates. One row was reserved for a negative control. Then, 50 μL of four-fold concentrated MICA, DOX and EtOH were added to the first three columns of each plate to generate OS 1 and OS 2. The remainder of each plate was used for single drug and positive/negative controls. Each strain was challenged with each drug alone, including 0.0313 μg/mL MICA, 0.01565 μg/mL MICA, 20% EtOH, 10% EtOH, and 800 μg/mL DOX. Metabolic activity was calculated using the XTT reduction assay as described. Each prevention and treatment assay contained three technical replicates and was repeated three times independently.

#### *C*. *albicans* metabolic activity in a silicone disk model

Several solutions were tested for activity as a prevention or treatment of SC5314 biofilms on silicone disks. These solutions included 20% EtOH with 800 μg/mL DOX and either 0.0313 or 0.01565 μg/mL MICA, solutions with 10% EtOH, 800 μg/mL DOX and either 0.0313 or 0.01565 μg/mL MICA and a solution of 10% EtOH with 200 μg/mL DOX and 0.0313 μg/mL MICA. Finally, a solution of 10% EtOH with the lowest MICA and DOX concentrations tested in the 3D checkerboards was also used. Drug solutions were made using 1:2 dilutions as described for the 2D checkerboard assay. Medical-grade silicone (Invotec, Jacksonville, FL) was washed with mild detergent then rinsed with deionized water, cut into disks that fit the well diameter, autoclaved, then aseptically inserted into the bottom of each well. The plate was then UV sterilized for 15–20 minutes before the addition of cells, media and drug solutions.

#### *C*. *albicans* colony regrowth assay

Promising solutions which decreased metabolic activity by ≥ 98% in the 3D checkerboard assay were chosen in addition to positive and negative controls to study *C*. *albicans* reference strain SC5314 regrowth from silicone disks [40]. These solutions were: 20% EtOH with 800 μg/mL DOX and either 0.0313 or 0.01565 μg/mL MICA, 10% EtOH with 800 μg/mL DOX and 0.0313 or 0.01565 μg/mL MICA, 20% EtOH alone, 10% EtOH alone, and 10% EtOH with 0.0313 μg/mL MICA and 200 μg/mL DOX. Each solution was made as in the 3D checkerboard assay with the addition of a sterilized, medical-grade silicone disk inserted into each well. After 24-hour incubation with a drug combination, disks were washed three times with PBS and then added to 5 mL of Sabouraud’s dextrose broth. The disk was then sonicated for three minutes and incubated at 30°C for 24 hours with shaking at 250 rpm. Absorbance was measured at 600nm and then 100 μL of each culture was plated to Sabouraud’s dextrose agar. Plates were incubated at 30°C for an additional 24 hours. Highly turbid cultures, including the positive controls and mature biofilms treated with 10% EtOH alone and in every combination, were plated as 1:400 dilutions to obtain an accurate CFU count. The number of observed CFU was then multiplied by the dilution factor to obtain the actual CFU. The colony regrowth assays on silicone disks were performed twice independently.

#### Treatment of mature polymicrobial biofilm with an optimized combination solution

Mono- and polymicrobial biofilms were grown as previously described with minor modifications [40, 42]. Briefly, wells of a 96-well plate were fitted with sterilized silicone discs. Wells harboring monomicrobial biofilms were inoculated with 100 μL of 1×10^6^ cells/mL of either *C*. *albicans* or *S*. *aureus* in TSB-g. To this, 100 μL of sterile TSB-g was added for a total volume of 200 μL per well. For wells harboring polymicrobial biofilms, 100 μL of *C*. *albicans* and *S*. *aureus* adjusted cell suspensions in TSB-g were added per well. Control disks were inoculated with 200 μL of sterile TSB-g. Plates were incubated at 37°C for 24 hours to allow for mature biofilm development.

#### XTT reduction assay of mature polymicrobial biofilms

Following mature monomicrobial and polymicrobial biofilm development, silicone disks were washed three times with 200 μL sterile PBS to remove non-adherent cells. One group of disks was inoculated with TSB-g, while the other was challenged with TSB-g containing 20% EtOH, 0.01565 μg/mL MICA, and 800 μg/mL DOX (OS 1). Plates were returned to a 37°C incubator for 24 hours. The following day, disks were washed three times with 200 μL sterile PBS and incubated with 200 μL of XTT working reagent for two hours at 37°C protected from light. Absorbance was read in a plate reader at 490 nm (VersaMax, Molecular Devices, Sunnyvale, CA).

#### Polymicrobial colony regrowth assay

Regrowth assays were performed as described previously [40,42]. Biofilms were grown on silicone disks as described above. Disks were challenged with either TSB-g (control) or OS 1 and incubated for 24 hours at 37°C. After washing three times with 200 μL PBS, disks were transferred to sterile 50-ml conical tubes containing 5 ml of YPD supplemented with 2 μg/mL vancomycin (VAN, for disks harboring *C*. *albicans* biofilms) or TSB containing 2.5 μg/mL amphotericin B (AMB, for disks harboring *S*. *aureus* biofilms). In order to obtain both *C*. *albicans* and *S*. *aureus* counts individually, polymicrobial biofilms were grown in parallel, so that one biofilm disk was regrown in YPD containing VAN and the other was regrown in TSB containing AMB. This step was necessary to inhibit the growth of either organism in the nonselective medium because overgrowth of the non-targeted microbe may have confounded the results. Samples were sonicated, and the tubes were returned to 30°C (*C*. *albicans*) or 37°C (*S*. *aureus*) and incubated with shaking at 200 rpm for 20 hours. After incubation, the CFUs were enumerated on selective agar media as described above; the lower and upper limits of detection were approximately 10^2^ CFU/mL and 10^8^ CFU/mL, respectively.

### Calculations of fractional inhibitory concentrations

#### 2D checkerboards

Fractional inhibitory concentrations (FICs) were calculated for each combination of drugs: MICA+EtOH, MICA+DOX, DOX+EtOH. The FIC value was calculated by dividing the MIC_90_ or MIC_50_ (the minimum inhibitory concentration of each drug which produces ≤ 10 or ≤ 50% metabolic activity, respectively) of one drug when used in combination by the MIC_90_ or MIC_50_ of the same drug when used alone, FIC of drug A = [Drug A_combination_]/[Drug A_alone_] [52]. Both MIC_90_ and MIC_50_ were calculated for each two-drug combination. The summation of each FIC value, FICI = FIC of drug A + FIC of drug B, determined whether the combination of drugs produced a synergistic, indifferent or antagonistic effect. The combination was considered synergistic when the FICI was ≤ 0.5, indifferent when the FICI was > 0.5 to ≤ 4, and antagonistic when the FICI was > 4 [21]. When the highest concentration of drug tested did not produce a 90% or 50% decrease in metabolic activity, the highest concentration of drug tested was used in place of the MIC_90_ or MIC_50_ to calculate FICI according to published methods [21, 52].

#### 3D checkerboards

FICI calculations for three drugs in combination were calculated using the equation above with the addition of a third FIC term (FICI = FIC of drug A + FIC of drug B + FIC of drug C) and the expansion of the definition of synergy up to FICI ≤ 0.75 [53]. A FICI value in the > 0.75 to ≤ 4 range was considered indifferent and a FICI of > 4 was considered antagonistic.

### Statistical analyses

The metabolic activities of forming and mature biofilms treated with optimized solution candidates were compared to the controls using two-way multiple comparisons analysis of variance (ANOVA) followed by a Tukey comparison post-test. One-way ANOVA was used to analyze monomicrobial colony regrowth from silicone disks. A Kruskal-Wallis one-way ANOVA and Dunn’s multiple comparisons post-test was used to analyze polymicrobial colony regrowth. Differences between groups were considered significant at a *P* value of *<0*.*05*. Statistical analyses were performed using GraphPad Prism 5.0 (GraphPad Software, Inc., San Diego, CA).

## Results

### 2D checkerboard assays

An indifferent effect was found in all two-drug combinations for both prevention and treatment of *C*. *albicans* monomicrobial biofilms when MIC_90_ (Table 1) and MIC_50_ (Table 2) criteria were utilized. Generally, all FICI values were approximately equal to one, except the combination of 5% (v/v) EtOH and 1.5626 μg/mL DOX, which had a FICI value approaching, but not meeting, the criteria for synergy (Table 1).

**Table 1:** Antifungal activity of two-drug combinations against forming and mature *C*. *albicans* biofilms, sMIC90.

**Biofilm**	Agent^a^	sMIC90^b^ of Each Drug		FICI^c^	**Outcome**
		**Alone**	**Combination**		
Prevention	MICA	0.01565 μg/mL	0.01565 μg/mL	1.031	Indifference
	EtOH (v/v)	10%	0.3125%		
	MICA	0.01565 μg/mL	0.01565 μg/mL	1.004	Indifference
	DOX	400 μg/mL	12.5 μg/mL		
	EtOH (v/v)	10%	5%	0.502	Indifference
	DOX	800 μg/mL	1.5625 μg/mL		
Treatment	MICA	0.0313 μg/mL	0.0313 μg/mL	1.031	Indifference
	EtOH (v/v)	20%	0.3125%		
	MICA	0.0313 μg/mL	0.0313 μg/mL	1.016	Indifference
	DOX	800 μg/mL	12.5 μg/mL		
	EtOH (v/v)	20%	20%	1.002	Indifference
	DOX	800μg/mL	mL		

a. micafungin (MICA); ethanol (EtOH); doxycycline (DOX)

b. Sessile minimum inhibitory concentration (sMIC90) is the concentration of each drug alone or in combination which inhibited 90% of metabolic activity.

c. FICI ≤ 0.5 = synergy, 4 ≥ FICI > 0.5 = indifference, FICI > 4 = antagonism

**Table 2:** Antifungal activity of two-drug combinations against forming and mature *C*. *albicans* biofilms, sMIC50.

**Biofilm**	Agent^a^	sMIC50^b^ of Each Drug		FICI^c^	**Outcome**
		**Alone**	**Combination**		
Prevention	MICA	0.01565μg/mL	0.01565μg/mL	1.031	Indifference
	EtOH (v/v)	10%	0.3125%		
	MICA	0.01565μg/mL	6.11×10^−5^μg/mL	1.004	Indifference
	DOX	400μg/mL	400μg/mL		
	EtOH (v/v)	5%	0.625%	0.513	Indifference
	DOX	800 μg/mL	400 μg/mL		
Treatment	MICA	0.0313μg/mL	2.45×10^−4^ μg/mL	1.008	Indifference
	EtOH (v/v)	10%	10%		
	MICA	0.0313μg/mL	0.0313μg/mL	1.016	Indifference
	DOX	800μg/mL	12.5μg/mL		
	EtOH (v/v)	10%	10%	1.002	Indifference
	DOX	800μg/mL	1.5625μg/mL		

a. micafungin (MICA); ethanol (EtOH); doxycycline (DOX)

b. Sessile minimum inhibitory concentration (sMIC50) is the concentration of drug alone or in combination which inhibited 50% of metabolic activity.

c. FICI ≤ 0.5 = synergy, 4 ≥ FICI > 0.5 = indifference, FICI > 4 = antagonism

#### 3D checkerboard assays

The combination of 2.5% EtOH, 0.0000153 μg/mL MICA and 12.5 μg/mL DOX demonstrated synergistic activity when used to prevent *C*. *albicans* biofilm formation (Table 3). An indifferent effect was observed for all other three-drug combinations tested (Tables 3 and 4) for prevention and treatment of biofilm formation regardless of which MIC criterion was used (i.e. MIC_50_ or MIC_90_).

**Table 3:** Antifungal activity of three-drug combinations against forming and mature *C*. *albicans* biofilms, sMIC50.

**Biofilm**	Agent^a^	sMIC50^b^ of Each Drug		FICI^c^	**Outcome**
		**Alone**	**Combination**		
Prevention	MICA	0.0313μg/mL	1.53×10^−5^ μg/mL	0.516	Synergy
	EtOH	5%	2.5%		
	DOX	800 μg/mL	12.5 μg/mL		
Treatment	MICA	0.0313 μg/mL	1.53×10^−5^ μg/mL	1.008	Indifference
	EtOH	20%	10%		
	DOX	800 μg/mL	g/mL		

a. micafungin (MICA); ethanol (EtOH); doxycycline (DOX)

b. Sessile minimum inhibitory concentration (sMIC50) is the concentration of drug alone or in combination which inhibited 50% of metabolic activity.

c. FICI ≤ 0.75 = synergy, 4 ≥ FICI > 0.75 = indifference, FICI > 4 = antagonism

**Table 4:** Antifungal activity of three-drug combinations against forming and mature *C*. *albicans* biofilms, sMIC90.

Biofilm	Agent^a^	sMIC90^b^ of Each Drug		FICI^c^	Outcome
		Alone	Combination		
Prevention	MICA	0.0313 μg/mL	6.11×10^−5^ μg/mL	0.752	Indifference
	EtOH	10%	2.5%		
	DOX	800 μg/mL	400 μg/mL		
Treatment	MICA	0.0313 μg/mL	1.5×10^−5^ μg/mL	1.008	Indifference
	EtOH	20%	20%		
	DOX	800 μg/mL	g/mL		

a. micafungin (MICA); ethanol (EtOH); doxycycline (DOX)

b. Sessile minimum inhibitory concentration (sMIC90) is the concentration of drug alone or in combination which inhibited 90% of metabolic activity.

c) FICI ≤ 0.75 = synergy, 4 ≥ FICI > 0.75 = indifference, FICI > 4 = antagonism

#### *C*. *albicans* metabolic activity measured by XTT reduction in a silicone disk model

**Fig 1:**
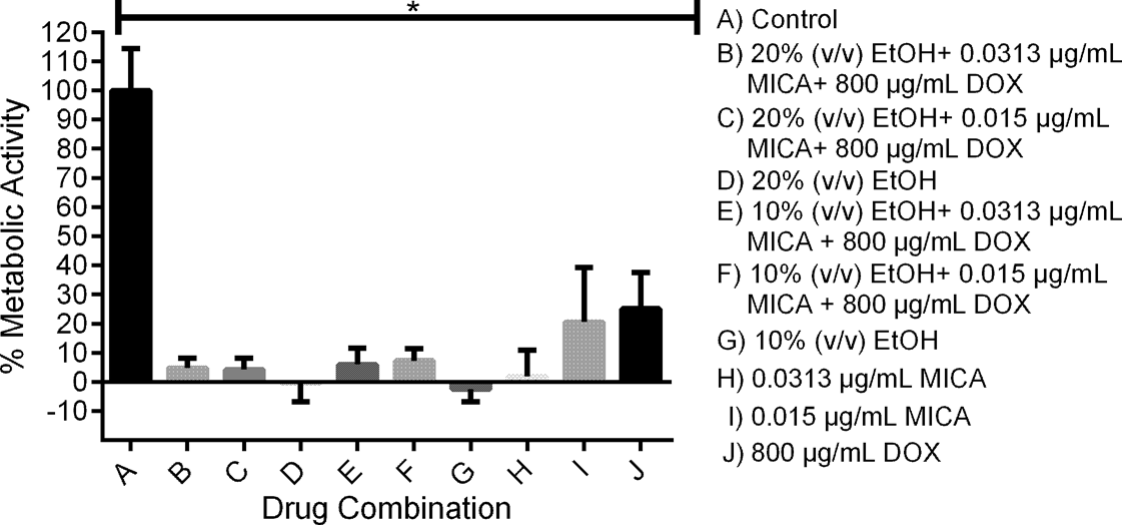
Prevention of *C*. *albicans* SC5314 biofilm formation on silicone disks. Potential lock solutions are highly effective in preventing the formation of *C*. *albicans* biofilms on silicone. Planktonic *C*. *albicans* cells were incubated on silicone for 24 hours at 37°C with potential lock solution candidates. Metabolic activity was measured using XTT reduction and untreated vs drug treated groups were compared using a one-way ANOVA. * *indicates p value of < 0*.*05*

**Fig 2:**
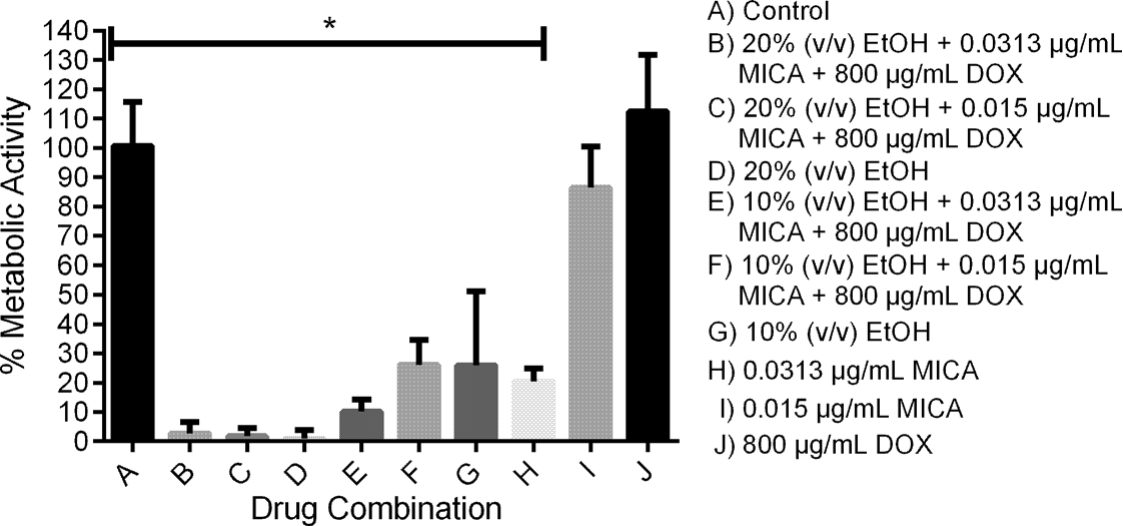
Treatment of mature SC5314 biofilms on silicone disks. Potential lock solutions significantly reduced metabolic activity of mature *C*. *albicans* biofilms. Biofilms were incubated on silicone for 24 hour at 37°C with potential lock solutions. Metabolic activity was measured using XTT reduction. Untreated and drug treated groups were compared using two-way ANOVA followed by Tukey post analysis. * *indicates p value of < 0*.*05*

Solutions with 20% EtOH, 800 μg/mL DOX and either 0.0313 or 0.01565 μg/mL MICA (MIC_90_ and MIC_50_ of MICA, respectively) as well as solutions with 10% EtOH, 800 μg/mL DOX and either 0.0313 or 0.01565 μg/mL MICA all reduced metabolic activity to less than five percent when used to prevent biofilm formation (Fig 1), except the combination of 10% EtOH with 0.01565 μg/mL MICA and 800 μg/mL DOX, which produced more than ten percent metabolic activity. When the combination solutions were tested against mature biofilms, they demonstrated a greater reduction in metabolic activity compared to MICA and DOX alone, but an equal reduction in metabolic activity compared to 20% EtOH alone (Fig 2).

#### *C*. *albicans* colony regrowth in a silicone disk model

**Fig 3:**
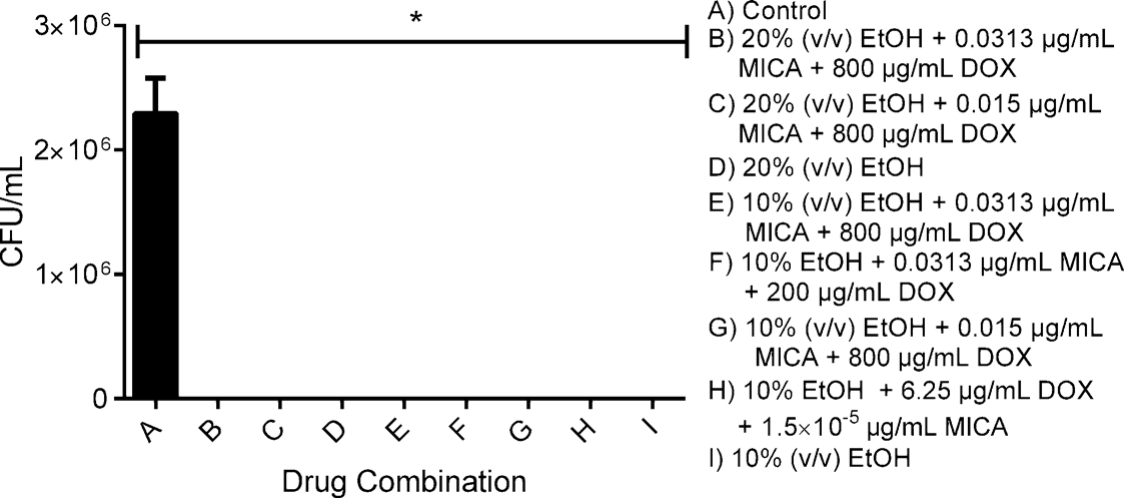
*C*. *albicans* SC5314 colony regrowth after 24 hour drug challenge. All combination solutions were highly effective in preventing the regrowth of forming biofilms on silicone. Following 24 hour drug challenge, forming biofilms were sonicated and incubated at 30°C for an additional 24 hours then plated on solid media to quantify regrowth. The untreated control was compared to the drug treated groups using one-way ANOVA. * indicates p value of < 0.05

**Fig 4:**
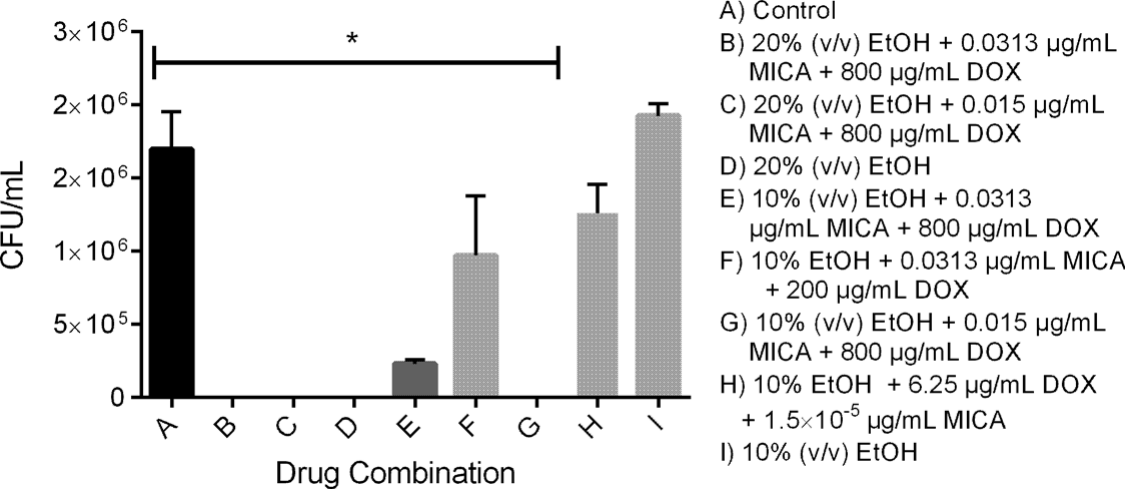
Mature *C*. *albicans* biofilm regrowth after 24 hour drug challenge. Combination solutions did not prevent the regrowth of mature biofilms on silicone. Following 24 hour drug challenge, biofilms were sonicated and incubated at 30°C for an additional 24 hours then plated solid media to quantify regrowth. Untreated and drug treated groups were compared using two-way ANOVA followed by Tukey post analysis. * indicates p value of < 0.05

The four combination solutions tested, as well as 20% and 10% EtOH alone were equally successful at preventing all *C*. *albicans* regrowth (Fig 3). Solutions containing 20% EtOH, either alone or in combination, prevented the regrowth of cells from mature biofilms (Fig 4). However, solutions containing 10% EtOH alone or in combination did not consistently eliminate fungal regrowth from mature biofilms.

#### Metabolic activity of optimized lock solutions against multiple *C*. *albicans* strains

**Fig 5:**
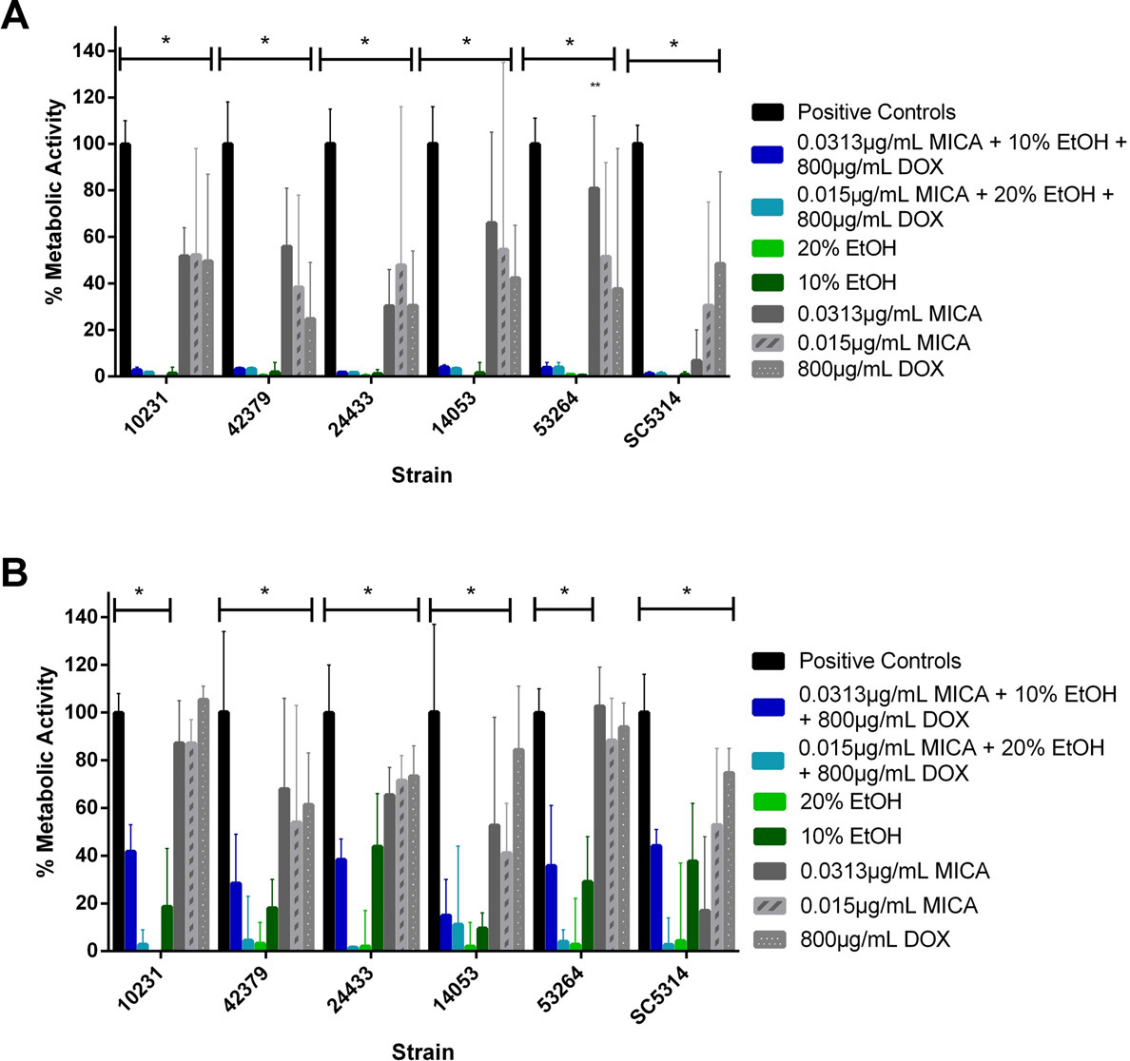
Prevention and treatment of clinical isolate and reference strain biofilm formation with optimized solutions. **Fig 5a.** Optimized solutions (OS 1, 20% ethanol, 0.015625 μg/mL micafungin, 800 μg/mL doxycycline, and OS 2, 10% ethanol with 0.0313 μg/mL micafungin and 800 μg/mL doxycycline) were highly effective in preventing the formation of echinocandin-resistant clinical isolate and reference strain biofilms. Optimized solutions were incubated with *C*. *albicans* isolates for 24 hours at 37°C to prevent biofilm development and untreated positive controls were compared to drug treated groups with the same strain. Metabolic activity was measured with XTT reduction. * *indicates p value of < 0*.*05*. **0.0313 μg/mL was not significant compared to the control for strain 53264 only. **Fig 5b.** Optimized solutions (OS 1, 20% ethanol, 0.015625 μg/mL micafungin, 800 μg/mL doxycycline, and OS 2, 10% ethanol with 0.0313 μg/mL micafungin and 800 μg/mL doxycycline) significantly reduce the metabolic activity of echinocandin-resistant clinical isolates and reference strains. Optimized solutions were incubated with mature *C*. *albicans* biofilms for 24 hours at 37°C. Metabolic activity was measured with XTT reduction and untreated positive controls were compared to drug treated groups with the same strain. * *indicates p value of < 0*.*05*

Two solutions selected from the preceding 3D checkerboard assays were further tested against all six strains (SC5314, ATCC10231, ATCC14053, ATCC24433, 42379, and 53264): 20% EtOH with 0.01565 μg/mL MICA and 800 μg/mL DOX (designated as “OS 1”), and 10% EtOH with 0.0313 μg/mL and 800 μg/mL DOX (designated as “OS 2”), (Prevention, Fig 5A; Treatment, Fig 5B). Across all strains, 20% EtOH alone was the most effective in preventing and treating mature biofilms, producing ≤ 1% metabolic activity for prevention and < 5% metabolic activity for treatment. Similarly, 10% EtOH alone was also highly effective, producing < 2% metabolic activity for prevention of biofilm formation and < 50% metabolic activity for treatment of mature biofilms. Using a Tukey comparison test, we observed a statistically significant decrease in metabolic activity between the no-drug control and both optimized solutions, across all strains for both prevention and treatment. No statistically significant difference in metabolic activity was found between 20% and 10% EtOH treatment groups when used alone or in combination (OS 1 vs. OS 2) to prevent biofilm formation. When used to treat a mature biofilm, a statistically significant difference was observed between OS 1 compared to OS 2, 20% EtOH compared to 10% EtOH, OS 1 compared to 20% EtOH, and OS 2 compared to 20% EtOH for strains 24433 and 53264. SC5314 was similar in that there was a statistically significant difference between the 20% and 10% EtOH alone, OS 1 and 20% EtOH, and OS 2 and 10% EtOH solutions. The optimized lock solutions were not more effective than 20% ethanol alone. This may be due to the increased activity of 20% vs 10% EtOH, and it is worth noting that there was no statistically significant difference between these solutions in the other three strains tested.

#### Metabolic activity of mature polymicrobial biofilms

**Fig 6:**
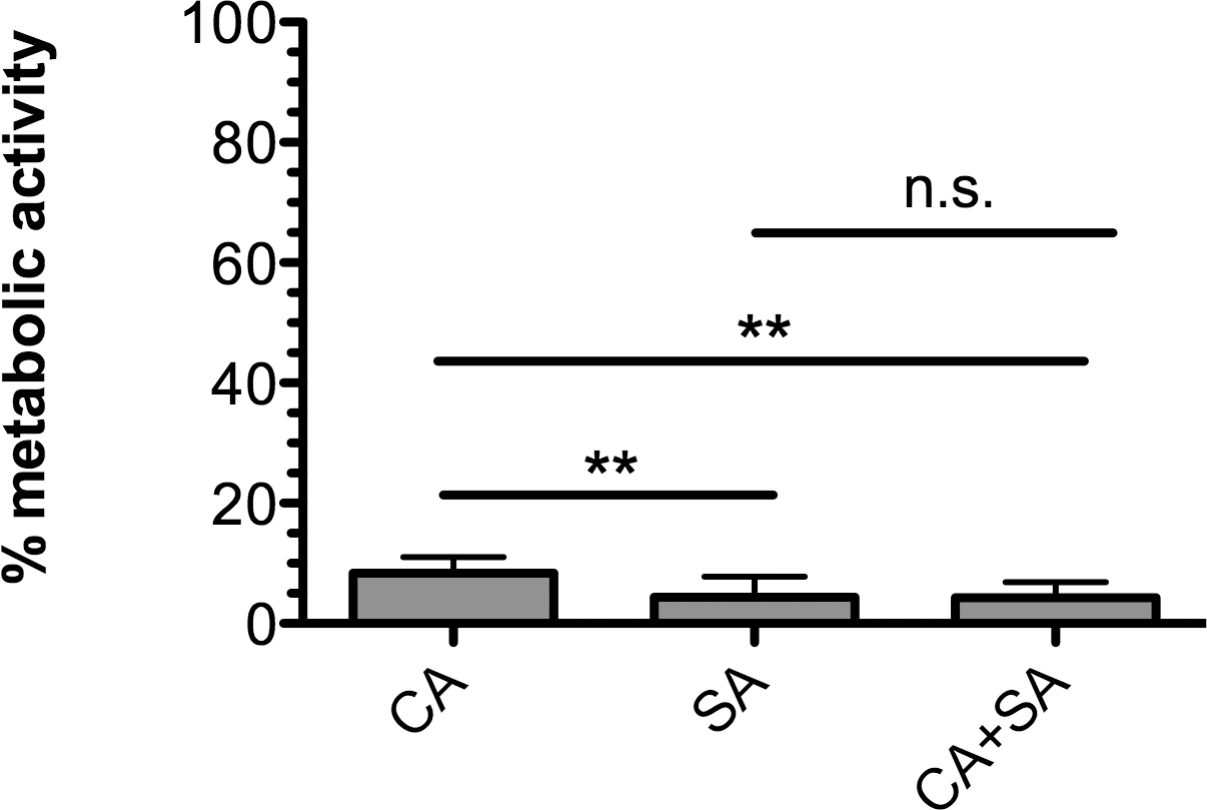
Treatment of mature *C*. *albicans* and *S*. *aureus* polymicrobial biofilms on silicone disks with OS 1. Monomicrobial (CA, SA) and polymicrobial (CA+SA) biofilms of *C*. *albicans* and *S*. *aureus* were challenged with drug free (DF) or optimized solution (OS 1, 20% ethanol, 800 μg/mL doxycycline, and 0.015625 μg/mL micafungin) prepared in sterile growth medium for 24 hours. Metabolic activity was assessed using the XTT assay and reported as % inhibition of the drug-free control. Error bars represent SDs. Data is cumulative of 4 independent experiments performed in triplicate. **, P ≤ 0.01, n.s., not significant using a one-way ANOVA and Tukey’s multiple comparisons post-test.

Incubation of mature polymicrobial (*C*. *albicans* + *S*. *aureus*) biofilms with OS 1 reduced the metabolic activity to less than 10% observed in control biofilms incubated with sterile media (Fig 6). There were no significant differences between metabolic activities of monomicrobial versus polymicrobial biofilms. Thus, OS 1 is highly effective at inhibiting proliferation of mature *S*. *aureus* and *C*. *albicans* mixed biofilms.

#### Polymicrobial regrowth in a silicone disk model

**Fig 7:**
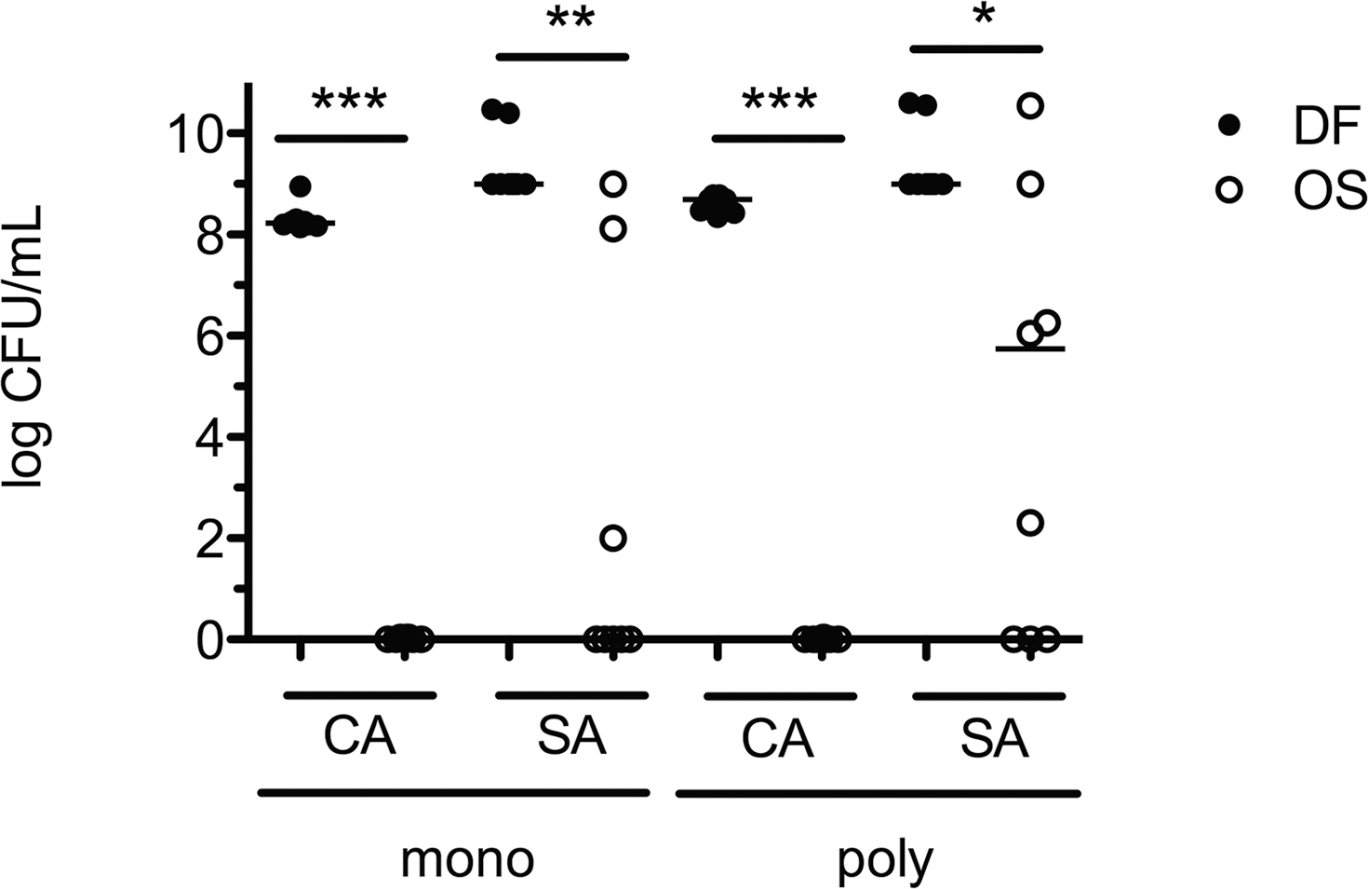
Polymicrobial regrowth after treatment with OS 1. Regrowth of mono- and polymicrobial biofilms on silicone disks after treatment with optimized lock solution. Monomicrobial (CA, SA) and polymicrobial (CA+SA) biofilms of *C*. *albicans* and *S*. *aureus* were challenged with drug free (DF, closed circles) or optimized solution (OS 1, 20% ethanol, 800 μg/mL doxycycline, and 0.015625 μg/mL micafungin, open circles) prepared in sterile growth medium for 24 hours. Immediately following lock solution treatment, disks were briefly sonicated and cultured in selective liquid medium for 24 hours. Serial dilutions were plated onto selective medium to quantify microbial regrowth. Horizontal line in dataset represents the median. Data is cumulative of four independent experiments performed in triplicate. *, P ≤ 0.05, ** P ≤ 0.01, *** P ≤ 0.001 using a Kruskal-Wallis one-way ANOVA and Dunn’s multiple comparisons post-test.

Optimized solution 1 (OS1) exhibited fungicidal activity by preventing regrowth of *C*. *albicans* in both monomicrobial and polymicrobial biofilms (Fig 7, CA, open circles) as compared to drug-free controls (Fig 7, CA, closed circles). Interestingly, despite significant reduction of metabolic activity observed in the XTT assay, regrowth of *S*. *aureus* was not completely inhibited in either monomicrobial or polymicrobial biofilms (Fig 7, SA, open circles). Staphylococcal regrowth potential was greater in polymicrobial biofilms (five of the eight disks) compared to monomicrobial biofilms (three of the eight disks); however, this difference was not statistically significant.

## Discussion

In this study, we demonstrate a combination solution that is highly effective against forming and mature *C*. *albicans* biofilms comprised of 20% EtOH, 0.01565 μg/mL MICA and 800 μg/mL DOX (OS 1), but was not more effective than 20% EtOH alone. This solution reduced the metabolic activity of forming biofilms to ≤ 2%, and prevented fungal regrowth from both forming and mature biofilms. These results suggest that OS 1 has potential for antifungal lock therapy and has a low ethanol concentration, therefore reducing the concern of structural damage to central venous catheters [28]. However, 20% EtOH alone produced the greatest reduction in clinical isolate and reference strain metabolic activity, both when used for prevention of biofilm formation and for treatment of mature biofilms. Additionally, OS 1 was effective in eliminating *C*. *albicans* from *C*. *albicans*- *S*. *aureus* polymicrobial biofilms and reduced *S*. *aureus* metabolic activity to <10%, although the solution did not demonstrate complete bactericidal activity. This is unsurprising given that the activity of DOX is dependent on cell viability and cells within the biofilm are metabolically inert.

Each component of the lock solution was carefully selected. Preliminary experiments in our laboratory demonstrated a ≥ 50% reduction in metabolic activity of sessile SC5314 when 0.0313 μg/mL MICA is used (see S1 File, MICA only experiments), which is consistent with prior studies in the literature. Studies which measured the susceptibility of invasive clinical isolates in planktonic form have reported MIC_90_s of ≤ 0.03 μg/mL [54–55] and MIC_50_s of ≤ 0.015 for MICA [55–56]. A number of other studies have reported similar MICs [29, 57–59]. AfLT employs supra-therapeutic concentrations of antifungals [4] and a concentration of 5 μg/mL has demonstrated success in an *in vitro* lock model [39]. However, the potent fungicidal activity of MICA, particularly when used in combination with EtOH, did not warrant a greater dose. Concentrations of EtOH and DOX were chosen for 3D checkerboard assays based on established single-drug activity against *C*. *albicans* [35, 40] and the 2D checkerboard assays. Furthermore, the activity of two and three drugs in combinations was investigated for synergistic activity by decreasing concentrations of each component. Synergistic activity was found with low dose MICA in combination with low doses of EtOH and DOX (Table 3), although such a combination would be ineffective as a lock solution.

The synergistic activity we observed with low concentrations of MICA, DOX and EtOH may be due to the combined activity of EtOH and DOX only, which together produced a FICI close to synergy (0.502, Table 1). With the addition of a third drug the FICI definition of synergy increases to include values ≤ 0.75. Given the extremely low concentration of MICA in the solution that produced synergy, we may be observing only the indifferent activity of EtOH and DOX combined. However, in the 2D checkerboards, a four-fold decreased concentration of EtOH is required to produce an MIC_50_ in combination compared to the MIC_50_ of EtOH alone (FIC_EtOH_ = 0.0125). When all three drugs are used in combination, there is a six-fold decrease in the amount of DOX required for 50% metabolic activity inhibition when used in a three-drug combination compared to the MIC_50_ of DOX alone (FIC_DOX_ = 0.0156). The concentration of the second drug (either EtOH or DOX) in the 2D and 3D checkerboards is halved when used in combination vs. alone. The decrease between FIC terms in two and three drug combinations suggests the activity is synergistic.

We chose a three-drug approach to AfLT to maximize the broad-spectrum activity of the solution to include yeasts and bacteria, to reduce the theoretical concern for development of resistance, and to minimize the ethanol concentration used. Even in the event that a catheter infection is resistant to one component of a lock solution, in principle, the other drugs should be effective, although this has yet to be tested. AfLT is not without risks and the selection of antimicrobial components should be carefully made. Concerns surrounding the use of a lock solution include the compatibility and stability of the selected agents as well as the effect on catheter integrity [60].

Limited investigation has been performed on the effect of high concentrations of ethanol to central venous catheters, including polyurethane and silicone. One study reported that 70% EtOH had no significant effect on the clinical performance of either catheter type after up to ten weeks of exposure [61]. In another study, silicone catheters exposed to 95% EtOH for 15 days showed no lumen degradation as assessed by scanning electron microscopy [62] A recent review identified reports describing the elution of 1,4-butanediol from polyurethane catheters and minor structural alterations to carbothane catheters after prolonged exposure to concentrations of ethanol greater than 60% or 30%, respectively [28]. A statistically significant decrease in force at break and elongation at break were observed for carbothane catheters exposed to 30% EtOH for nine weeks. However, the change in force and elongation at break was not significant enough to prevent clinical utility [63]. While not explicitly tested in catheter integrity studies, the concentration of ethanol required for *C*. *albicans* sterilization in this study are well below the concentrations implicated in catheter damage, suggesting that the EtOH component of our proposed solution is less likely to affect catheter integrity.

These data demonstrate a highly effective optimized solution (20% EtOH, 0.01565 μg/mL MICA, 800 μg/mL DOX, OS 1), which prevents the formation of *C*. *albicans* biofilms, but was not more effective than 20% EtOH alone. Our study is limited by the static nature of biofilm prevention and treatment assays and the lack of physiological conditions in this *in vitro* model. The activity of EtOH, MICA and DOX in combination against polymicrobial biofilms is the subject of ongoing investigation. Further investigation of the clinical utility of other combination solutions is warranted.

## Supporting Information

S1 File Biofilm metabolic activity data for 2D and 3D checkerboard experiments, used to generate values given in Tables 1–4. (XLSX)

S2 File Biofilm metabolic activity and CFU data, summarized graphically in Figs 1–7. (XLSX)
